# A Sea Buckthorn Pomace Polysaccharide Mitigates H_2_O_2_-Induced Impairment of Intestinal Barrier Integrity in Caco-2 Monolayers

**DOI:** 10.3390/foods15152591

**Published:** 2026-07-24

**Authors:** Jinmei Zhao, Huan Miao, Fang Wu, Juan Wei

**Affiliations:** 1 College of Life Sciences and Engineering, Hexi University, Zhangye 734000, China; 15109313733@163.com (H.M.); 18119375469@163.com (F.W.); 2 College of Food Science and Engineering, Gansu Agricultural University, Lanzhou 730070, China; weijuan@gsau.edu.cn

**Keywords:** polysaccharides, *Hippophae rhamnoides* subsp. *sinensis*, intestinal barrier, tight junctions, oxidative stress

## Abstract

Oxidative stress is known to disrupt intestinal barrier integrity, whereas certain polysaccharides have demonstrated protective effects on intestinal function. This study aimed to isolate a polysaccharide from processed *Hippophae rhamnoides* subsp. *sinensis* pomace (remaining after oil and juice extraction) and evaluate its protective effect against H_2_O_2_-induced intestinal barrier dysfunction in Caco-2 cell monolayers. A polysaccharide fraction, designated SPP-0.2b, with a weight-average molecular weight (Mw) of 42.405 kDa, was successfully isolated by hot water extraction and purified using DEAE-52 cellulose and Sephadex G-100 gel chromatography. Monosaccharide composition analysis revealed that SPP-0.2b was mainly composed of rhamnose, arabinose, galactose, mannose, and galacturonic acid. Compared with the H_2_O_2_-treated group, SPP-0.2b (500 μg/mL) increased the transepithelial electrical resistance (TEER) by 76.67% and reduced basolateral FITC-dextran fluorescence intensity by 34.63% in Caco-2 monolayers. In addition, SPP-0.2b significantly increased the protein expression levels of Occludin and Zonula Occludens-1 by 79.49% and 66.75%, respectively. SPP-0.2b also reduced intracellular reactive oxygen species and malondialdehyde levels while enhancing the activities of superoxide dismutase and glutathione peroxidase. Collectively, these findings suggest that the protective effect of SPP-0.2b against H_2_O_2_-induced intestinal barrier dysfunction is associated with the maintenance of tight junction proteins and attenuation of oxidative stress. This study provides experimental evidence supporting the further investigation of SPP-0.2b as a potential functional food ingredient for intestinal health.

## 1. Introduction

The intestinal epithelial barrier serves as the first line of natural defense in the gastrointestinal tract. It forms the structural basis for the intestine’s selective permeability, enabling the passive absorption of luminal nutrients, ions, and water while restricting the translocation of microorganisms and pathogenic antigens [1]. The intestinal epithelium consists of a continuous monolayer of epithelial cells interconnected by specialized junctional complexes [2]. Among these, tight junctions (TJs), located at the apical region and lateral borders of intestinal epithelial cells, are essential for maintaining the mechanical barrier function of the intestinal mucosa. By sealing intercellular spaces, TJs tightly connect adjacent cells and regulate paracellular permeability [3]. It is well established that excessive accumulation of reactive oxygen species (ROS) can impair intestinal barrier function by inducing oxidative stress and destabilizing tight junctions [4]. Various factors, including alcohol consumption, pharmaceuticals, and certain food components, may trigger excessive ROS production in the intestine, leading to epithelial barrier disruption and consequently contributing to numerous health disorders [5].

Polysaccharides are naturally occurring macromolecular carbohydrates widely distributed in microorganisms, plants, and animals [6]. They are generally composed of monosaccharide units linked by glycosidic bonds and exhibit considerable structural diversity, complexity, and broad molecular weight distributions ranging from tens of thousands to several million Daltons [7]. Numerous studies have demonstrated that polysaccharides possess diverse biological activities, including immunomodulatory, anti-aging, antibacterial, and anticancer properties, many of which are closely associated with their antioxidant capacity [8]. Recent studies have further shown that certain polysaccharides can preserve intestinal barrier integrity by alleviating oxidative stress and regulating tight junction function. For example, Li et al. [9] reported that polysaccharides extracted from *Plantago asiatica* L. alleviated nonylphenol-induced increases in paracellular permeability in Caco-2 monolayers by upregulating TJ proteins, including zonula occludens-1 (ZO-1), Claudin-1, and Occludin. Similarly, Li et al. [10] demonstrated that microalgal polysaccharides protected intestinal epithelial barrier function by attenuating intracellular ROS accumulation and TJs disruption in both H_2_O_2_-treated Caco-2 cells and dextran sulfate sodium-induced colitis mouse models.

Sea buckthorn was originally used for soil and water conservation in China, and the country accounts for more than 90% of global production [11]. As both an important traditional medicinal and edible plant, sea buckthorn berries are abundant in functional components, including carotenoids, phenolics, organic acids, and vitamins [12]. These berries are commonly processed for oil extraction and juice production. However, the resulting pomace is often discarded or underutilized. Notably, sea buckthorn pomace remains rich in bioactive constituents, among which polysaccharides are considered one of the major functional components. Previous studies have reported that polysaccharides from sea buckthorn exhibit antioxidant, hepatoprotective, and anti-inflammatory activities [13,14,15].

Although several studies have reported the extraction methods, monosaccharide compositions, molecular weights, and biological activities of sea buckthorn polysaccharides, these characteristics vary considerably depending on the species, plant tissues, and geographical origins of the raw materials [16]. Chinese sea buckthorn (*Hippophae rhamnoides* subsp. *sinensis*) is the most widely distributed and highest-yielding wild subspecies in China, with the Qinghai–Tibetan Plateau recognized as both its center of origin and its primary natural habitat [17]. To the best of our knowledge, previous studies on sea buckthorn polysaccharides have mainly focused on polysaccharides extracted from sea buckthorn berries [13,14,15]. In contrast, little attention has been paid to polysaccharides recovered from sea buckthorn pomace, a major by-product generated during oil and juice processing. Moreover, the intestinal barrier-protective activity of sea buckthorn pomace polysaccharides has not yet been systematically investigated.

Therefore, in the present study, a polysaccharide fraction, designated SPP-0.2b, was isolated and purified from the pomace of *H. rhamnoides* subsp. *sinensis* obtained after oil and juice extraction. Its molecular weight and monosaccharide composition were characterized by high-performance gel permeation chromatography (HPGPC) and ion chromatography, respectively. Furthermore, the protective effects of SPP-0.2b against H_2_O_2_-induced intestinal barrier dysfunction were evaluated using a Caco-2 cell monolayer model. This study provides new insights into the value-added utilization of sea buckthorn processing by-products and expands current knowledge regarding the biological activities of sea buckthorn polysaccharides.

## 2. Materials and Methods

### 2.1. Materials

Chinese sea buckthorn (*Hippophae rhamnoides* subsp. *sinensis*) berries were harvested in October and identified according to the Flora of China. Pomaces were obtained from Gansu Gannong Biotechnology Co., Ltd. (Lanzhou, China) following the extraction of oil and juice.

### 2.2. Reagents

DEAE-Cellulose 52 was purchased from YuanYe Biotechnology Co., Ltd. (Shanghai, China). Sephadex G-100 was provided by GE Healthcare Biosciences (Pittsburgh, PA, USA). Dextran standards and monosaccharide standards were obtained from Sigma-Aldrich (St. Louis, MO, USA). Minimum Essential Medium (MEM) and fetal bovine serum (FBS) were obtained from Servicebio Technology Co., Ltd. (Wuhan, China). BCA protein assay kit (WLA004), Malondialdehyde (MDA) assay kit (WLA048), glutathione peroxidase (GSH-Px) assay kit (WLA107), superoxide dismutase (SOD) assay kit (WLA110), rapid preparation for SDS-PAGE gel (WLA013), electrochemiluminescence (ECL) detection reagents were purchased from Wanleibio Co., Ltd. (Shenyang, China). Cell Counting Kit-8 (CCK-8) were purchased from BioTeke Corporation (Beijing, China).

### 2.3. Extraction, Isolation, and Purification of Sea Buckthorn Pomace Polysaccharides

Sea buckthorn pomace polysaccharides were extracted following the method of Zhang et al. [18]. Briefly, pomace powder was extracted by boiling water (3 h × 3), deproteinized with the Sevag method, and precipitated by four volumes of ethanol overnight. Precipitate was freeze-dried to obtain crude polysaccharides (yield: 3.7 g/100 g).

Crude polysaccharides (30 g) were fractionated by DEAE-52 cellulose column, eluted with MiliQ water, 0.2, 0.5, and 1.0 mol/L NaCl. Fractions were monitored by phenol-sulfuric acid method, collected, dialyzed, and freeze-dried. The obtained sea buckthorn pomace polysaccharide (SPP) fractions were designated as SPP-0, SPP-0.2, SPP-0.5, and SPP-1.0, respectively, and their yields were calculated.

SPP-0.2 was further purified using Sephadex G-100 automated gel chromatography purification system (BRT-GS, BoRui Saccharide Biotech Co., Ltd., Yangzhou, China) with online refractive index detection. Purified fractions were designated SPP-0.2a, SPP-0.2b, SPP-0.2c, and SPP-0.2d.

### 2.4. Molecular Weight Determination of Polysaccharide Fractions

Molecular weights were determined by HPGPC according to Chen et al. [19] using a Shimadzu LC-10A system with RI-10A detector (Shimadzu Corporation, Kyoto, Japan). Samples (5 mg/mL) were separated on a tandem gel column (BRT105-104-102; BoRui Saccharide, Yangzhou, China) with 0.05 mol/L NaCl as mobile phase (0.6 mL/min, 40 °C).

### 2.5. Monosaccharide Composition Analysis of SPP-0.2b

Monosaccharide composition of SPP-0.2b was determined by ion chromatography (ICS5000, Thermo Fisher, Waltham, MA, USA) according to Liu et al. [20]. Samples were hydrolyzed with 3 mol/L C_2_HF_3_O_2_ (120 °C, 3 h), dried under nitrogen, and resuspended for analysis. Separation was performed on a Dionex CarboPac™ PA20 column (3 × 150 mm) (Dionex, Sunnyvale, CA, USA). The mobile phase consisted of solvent A (H_2_O) and solvent B (15 mmol/L NaOH). The flow rate was 0.3 mL/min, the injection volume was 5 μL, and the column temperature was maintained at 30 °C.

### 2.6. Cell Culture

Caco-2 cells (CSTR:19375.09.3101HUMTCHu146) were obtained from the National Collection of Authenticated Cell Cultures (Shanghai, China) and cultured in MEM with 1% penicillin–streptomycin solution and 10% FBS at 37 °C with 5% CO_2_. When cell confluence reached approximately 80%, cells were passaged for further experiments.

### 2.7. Cell Cytotoxicity Analysis

Cytotoxicity was assessed by the CCK-8 assay according to Ling et al. [21]. Caco-2 cells (1 × 10^5^ cells/mL) were seeded and treated for 24 h with SPP-0.2b at final concentrations of 10–500 µg/mL. The 450-nm absorbance values were recorded via a spectrophotometer (Bio-Rad Laboratories, Inc., Hercules, CA, USA) after exposing the cells to CCK-8 fluid for a 4-h duration.

Cell viability was calculated following equation: (As–Ab)/(Ac – Ab) × 100%.

As: absorbance of the treatment group; Ac: absorbance of the control group; Ab: absorbance of the blank group.

### 2.8. Measurement of Transepithelial Electrical Resistance (TEER) Value and Fluorescein Isothiocyanate-Dextran (FITC-Dextran) Fluorescence Intensity

Following the method described by Chen and Kitts [22], Caco-2 cells were seeded onto Transwell^®^ inserts (24-well plates, Corning, NY, USA) and cultured for 21 days to establish differentiated monolayers. Monolayers with TEER > 500 Ω·cm^2^ (measured by ERS-2, Merck Millipore) underwent a 2-h incubation with varied concentrations of SPP-0.2b (125, 250, 500 µg/mL), prior to 1 h of exposure to 250 μmol/L H_2_O_2_ [23,24]. TEER was re-measured and expressed as percentage of initial value.

To evaluate paracellular permeability, FITC-dextran (4 kDa; Sigma-Aldrich, St. Louis, MO, USA) was dissolved in serum-free MEM at a final concentration of 1 mg/mL and added to the apical chamber (200 μL), while 600 μL of serum-free MEM was added to the basolateral chamber. After incubation for 4 h at 37 °C, 100 μL of medium was collected from the basolateral chamber, and the fluorescence intensity was measured using a Synergy™ HT multimode microplate reader (BioTek Instruments, Winooski, VT, USA) with excitation and emission wavelengths of 490 nm and 520 nm, respectively. The results are presented as basolateral FITC-dextran fluorescence intensity, with higher fluorescence intensity indicating increased paracellular permeability and impaired intestinal barrier integrity.

### 2.9. Western Blot Analysis

Caco-2 cell monolayers were pretreated with SPP-0.2b (125, 250, or 500 μg/mL) for 2 h, followed by exposure to 250 μmol/L H_2_O_2_ for 1 h, as previously described [23,24]. Groups treated only with H_2_O_2_ or SPP-0.2b served as the H_2_O_2_-induced damage group and the SPP-0.2b-only treatment group, respectively.

Total cellular protein was extracted using a Cell Lysis Kit. Briefly, 600 μL of lysis buffer supplemented with phenylmethylsulfonyl fluoride (PMSF) was added to each culture dish. After incubation on ice for 30 min, the lysates were centrifuged at 10,000× *g* for 10 min, and the supernatants containing total protein were collected. Protein concentrations were determined using a BCA Protein Assay Kit.

Equal amounts of protein (40 μg) were separated by 10% SDS-PAGE and transferred onto polyvinylidene difluoride (PVDF) membranes (Merck Millipore, Burlington, MA, USA). The membranes were washed with Tris-Buffered Saline with Tween 20 (TBST) for 5 min and then blocked with 5% non-fat skim milk in TBST for 1 h at room temperature. Subsequently, the membranes were incubated overnight at 4 °C with primary antibodies against β-actin (WL01372, 1:1000), Occludin (WL01996, 1:500), and ZO-1 (WL03419, 1:500) (Wanleibio Co., Ltd., Shenyang, China). After washing with TBST, the membranes were incubated with an HRP-conjugated goat anti-rabbit IgG secondary antibody (WLA023, 1:5000; Wanleibio Co., Ltd., Shenyang, China) for 45 min at 37 °C.

Protein bands were visualized using enhanced chemiluminescence (ECL) reagents, and band intensities were quantified using Gel-Pro Analyzer software (Gel-Pro Analyzer 4.0). The expression levels of target proteins were normalized to β-actin and expressed as fold changes relative to the control group.

### 2.10. Assessment of ROS

According to Wei et al. [25], Caco-2 cells were treated and grouped with H_2_O_2_ and SPP-0.2b as described above. Then, cells from each group were collected and treated with 1 mL of DCFH-DA dilution (diluted 1:1000 with medium). After 20 min of incubating at 37 °C, cells were washed. A NovoCyte flow cytometer (ACEA Biosciences, San Diego, CA, USA), operating at excitation and emission wavelengths of 485 and 535 nm, respectively, was utilized to evaluate the fluorescence intensity of the cell suspensions.

### 2.11. Evaluation of MDA Content and GSH-Px, SOD Activity

According to Wei et al. [25], Caco-2 cells were treated with H_2_O_2_ and SPP-0.2b as described above. After that, cells were collected, washed and resuspended with PBS, then lysed by ultrasonication in an ice bath and centrifuged at 1500 *g* for 10 min. Protein of each group was quantified through BCA Protein Assay kit. The content of MDA was evaluated via MDA Assay Kit (nmol/mg prot). Furthermore, activities of GSH-Px and SOD were estimated via GSH-Px Assay Kit and SOD Assay Kit (U/mg prot).

### 2.12. Data Analysis

All statistical evaluations were performed using SPSS Statistics 26.0 software (IBM, Armonk, NY, USA). All experiments were independently performed three times, and the data are presented as mean ± standard deviation (SD) (*n* = 3), where *n* represents independent biological experiments. Significant differences were determined using Duncan’s multiple range tests, with *p* < 0.05 indicating statistical significance.

## 3. Results and Discussion

### 3.1. Isolation, Purification, and Molecular Weight Determination of SPP

#### 3.1.1. Isolation

**Figure 1:**
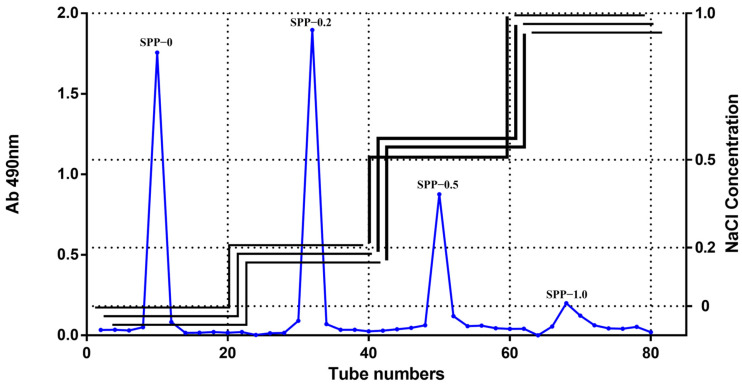
Elution curve of sea buckthorn pomaces polysaccharide (SPP) on DEAE-52 cellulose column.

As shown in Figure 1, the corresponding peaks were sequentially collected from left to right and freeze-dried to obtain polysaccharide fractions with different polarities, designated as SPP-0, SPP-0.2, SPP-0.5, and SPP-1.0, respectively. The masses of these fractions were 4.5, 5.0, 1.5, and 0.7 g, corresponding to yields of 15.0%, 16.6%, 5.0%, and 2.3%, respectively.

#### 3.1.2. Purification

**Figure 2:**
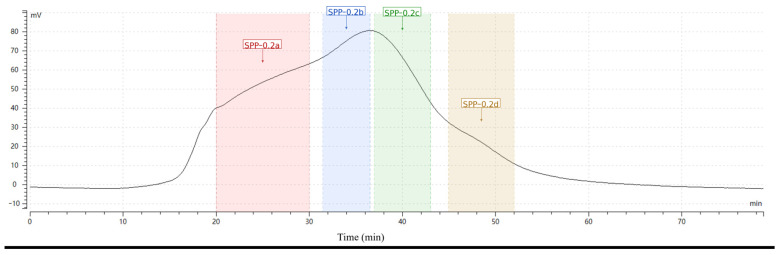
Sephadex G-100 automated gel chromatography purification profile of SPP-0.2.

The polysaccharide fraction SPP-0.2, which exhibited the highest yield, was further purified by Sephadex G-100 automated gel chromatography. As shown in Figure 2, the symmetrical portions of the first peak (20–30 min), second peak (31.5–36.5 min), third peak (37–43 min), and fourth peak (45–52 min) were separately collected and designated as SPP-0.2a, SPP-0.2b, SPP-0.2c, and SPP-0.2d, with yields of 14.4%, 5.6%, 6.4%, and 1.5%, respectively.

#### 3.1.3. Molecular Weight Determination

**Figure 3:**
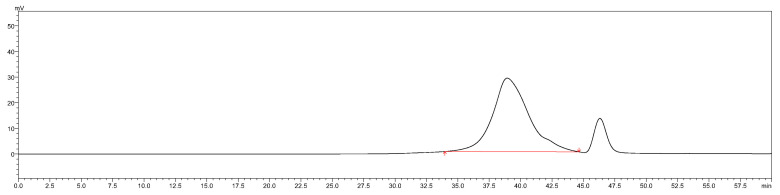
HPGPC chromatograms of SPP-0.2b for molecular weight determination.

The molecular weights of SPP-0.2a, SPP-0.2b, SPP-0.2c, and SPP-0.2d were determined by HPGPC. The results showed that only SPP-0.2b exhibited a single symmetrical peak, indicating a relatively homogeneous polysaccharide fraction. The weight-average molecular weight (Mw), number-average molecular weight (Mn), and peak molecular weight (Mp) of SPP-0.2b were 42.405 kDa, 28.935 kDa, and 34.575 kDa, respectively. The polydispersity index (PDI, Mw/Mn) was 1.47, indicating a relatively narrow molecular weight distribution (Figure 3). Therefore, SPP-0.2b was selected for subsequent investigations, including monosaccharide composition analysis and evaluation of its intestinal protective activity.

### 3.2. Monosaccharide Composition of SPP-0.2b

**Figure 4:**
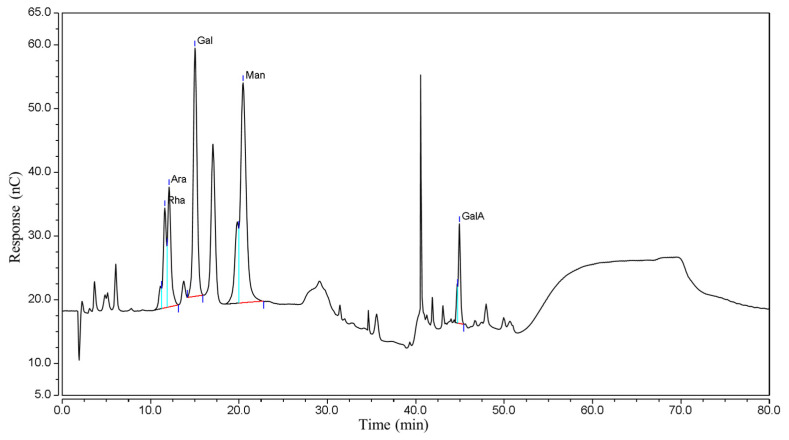
Ion chromatogram of monosaccharide composition of SPP-0.2b.

The ion chromatography results are shown in Figure 4. SPP-0.2b was mainly composed of rhamnose (Rha, 140.43 μg/mg), arabinose (Ara, 62.90 μg/mg), galactose (Gal, 216.87 μg/mg), mannose (Man, 374.28 μg/mg), and galacturonic acid (GalA, 58.47 μg/mg). Based on the presence of GalA, SPP-0.2b was considered to be a weakly acidic heteropolysaccharide.

### 3.3. Protective Effect of SPP-0.2b on the Barrier Integrity of Caco-2 Monolayers

The cytotoxicity assay demonstrated that SPP-0.2b at concentrations ≤ 500 μg/mL exerted no significant influence on Caco-2 cell viability, indicating that SPP-0.2b exerted no apparent cytotoxic effects on Caco-2 cells (Figure S1, Supplementary Materials). Therefore, subsequent experiments utilized 125, 250, and 500 μg/mL of SPP-0.2b as the low, medium, and high concentrations, respectively.

**Figure 5:**
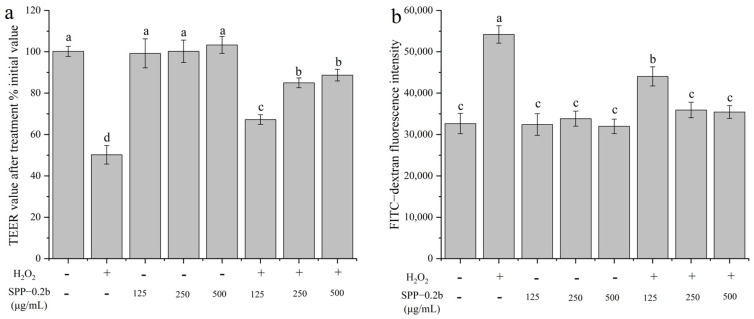
Protective effect of SPP-0.2b on the barrier integrity of Caco-2 monolayers. (**a**) SPP-0.2b restored H_2_O_2_-induced decreasing of TEER value; (**b**) SPP-0.2b restored H_2_O_2_-induced increasing of basolateral FITC-dextran fluorescence intensity. Data are shown as mean ± SD (*n* = 3). Different letters represent significant difference (*p* < 0.05).

TEER and FITC-dextran basolateral fluorescence intensity are widely used indicators of intestinal barrier integrity and epithelial permeability [21]. As shown in Figure 5a,b, H_2_O_2_ treatment significantly decreased the TEER values and increased the basolateral FITC-dextran fluorescence intensity in Caco-2 monolayers, indicating disruption of barrier integrity and increased epithelial permeability. In contrast, pretreatment with SPP-0.2b dose-dependently attenuated the detrimental effects of H_2_O_2_ on Caco-2 monolayers, as evidenced by increased TEER values and reduced basolateral FITC-dextran fluorescence intensity. At a concentration of 500 μg/mL, SPP-0.2b increased TEER values by 76.67% and reduced the basolateral FITC-dextran fluorescence intensity by 34.63% compared with the H_2_O_2_-treated group (Figure 5a,b). These findings demonstrated that SPP-0.2b effectively protected the integrity of Caco-2 cell monolayers against H_2_O_2_-induced barrier disruption.

Our findings are consistent with previous studies reporting the protective effects of natural polysaccharides on intestinal barrier function. For example, polysaccharides from *Oudemansiella raphanipes* alleviated D-galactose-induced damage to Caco-2 monolayer integrity by increasing TEER values [26]. Similarly, a polysaccharide isolated from *Acmella oleracea* leaves significantly decreased basolateral FITC-dextran fluorescence intensity in IL-1β-treated Caco-2 monolayers, thereby maintaining epithelial barrier integrity [27].

### 3.4. SPP-0.2b Alleviates H_2_O_2_-Induced Reduction of Occludin and ZO-1 Proteins

**Figure 6:**
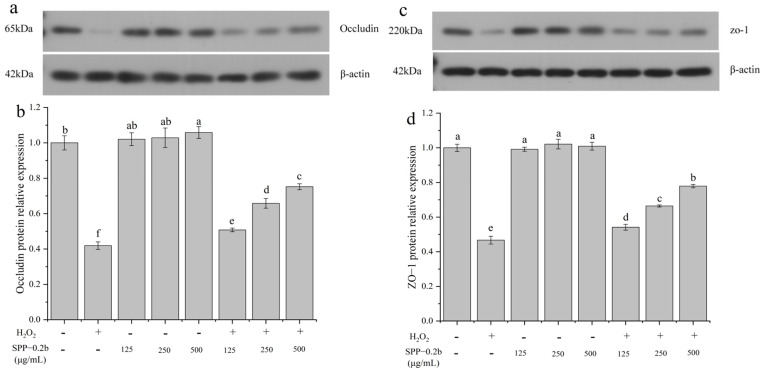
SPP-0.2b alleviates H_2_O_2_-induced reduction of Occludin and ZO-1 proteins in Caco-2 cells. (**a**,**c**) Representative Western blot images of Occludin and ZO-1 proteins. (**b**,**d**) Relative protein levels of Occludin and ZO-1. Protein expression was normalized to β-actin and presented as fold change relative to the control group. Data are shown as mean ± SD (*n* = 3). Different letters represent significant difference (*p* < 0.05).

The structural cohesion and permeability barrier of TJs heavily rely on Occludin, a distinctive four-pass transmembrane protein. Furthermore, a cytoplasmic scaffolding protein, ZO-1, links Occludin to the actin cytoskeleton, contributing to TJs stability [28]. As shown in Figure 6, H_2_O_2_ treatment markedly inhibited protein content of Occludin and ZO-1 by 58.19% and 53.28%, respectively. Oxidative stress induced by H_2_O_2_ is considered a key factor contributing to TJs disruption in the intestinal barrier, involving both altered expression and post-translational modification of TJ proteins [4]. Pretreatment with SPP-0.2b dose-dependently alleviated the H_2_O_2_-induced downregulation of Occludin and ZO-1. At a concentration of 500 μg/mL, SPP-0.2b increased the protein expression levels of Occludin and ZO-1 by 79.49% and 66.75%, respectively, compared with H_2_O_2_-treated cells (Figure 6). These results indicate that SPP-0.2b ameliorated H_2_O_2_-induced intestinal barrier dysfunction, at least in part, by upregulating the expression of TJ proteins. Similar protective effects have been reported for other natural polysaccharides. For instance, microalgal polysaccharides and Huaier polysaccharides exhibited protective effects against H_2_O_2_- or DSS-induced intestinal barrier disruption in Caco-2 cells and mouse colitis models by enhancing TJs protein expression [10,29]. Therefore, the upregulation of Occludin and ZO-1 may contribute to the intestinal barrier protective effect of SPP-0.2b.

### 3.5. SPP-0.2b Attenuates H_2_O_2_-Induced ROS Accumulation and MDA Elevation

**Figure 7:**
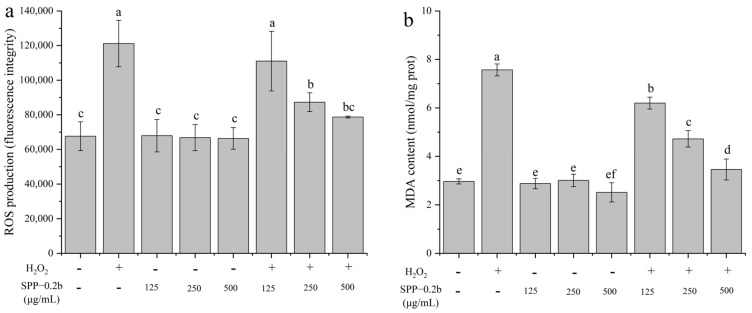
SPP-0.2b attenuates H_2_O_2_-induced accumulation of (**a**) ROS and (**b**) MDA in Caco-2 cells. Data are shown as mean ± SD (*n* = 3). Different letters represent significant difference (*p* < 0.05).

Oxidative stress in intestine is a major contributor to intestinal injury and serves as a key trigger for intestinal barrier dysfunction [5]. Song and Gao [24] reported that H_2_O_2_ treatment disrupted intestinal barrier integrity by inducing excessive intracellular ROS production. Similar findings were observed in the present study, in which treatment with 250 μmol/L H_2_O_2_ significantly increased intracellular ROS levels to 1.79-fold of those in the control group (Figure 7a). Pretreatment with SPP-0.2b significantly attenuated H_2_O_2_-induced ROS accumulation in a concentration-responsive manner. Notably, the highest tested dose of SPP-0.2b (500 µg/mL) reduced ROS levels by 35.06% compared with the H_2_O_2_-treated group, whereas treatment with SPP-0.2b alone had no significant effect on ROS production (Figure 7a).

Due to the high reactivity of ROS toward unsaturated fatty acids in phospholipid bilayers, lipid peroxidation is an inevitable consequence of oxidative stress. Lipid peroxidation can severely impair normal cellular functions by altering membrane structure, fluidity, and permeability [30]. MDA, a major end-product of lipid peroxidation, is commonly used as a biomarker of oxidative damage and membrane lipid peroxidation [31]. In the present study, H_2_O_2_ treatment increased intracellular MDA levels to 2.55-fold of those in the control group. However, pretreatment with 125, 250, and 500 μg/mL SPP-0.2b reduced MDA accumulation by 18.11%, 37.59%, and 54.34%, respectively, compared with the H_2_O_2_-treated group (Figure 7b). Consistent with our findings, peach gum polysaccharides were also reported to alleviate oxidative stress by normalizing SOD activity and MDA levels [32]. Accumulating evidence suggests that excessive ROS generation and MDA accumulation contribute to intestinal barrier disruption [33]. Therefore, suppression of ROS overproduction and lipid peroxidation may contribute to the intestinal barrier protective effect of SPP-0.2b against H_2_O_2_-induced dysfunction.

### 3.6. SPP-0.2b Reverses H_2_O_2_-Induced Suppression of Antioxidant Defense System

To maintain redox homeostasis, cells possess an intrinsic antioxidant defense system composed of both enzymatic and non-enzymatic antioxidants. SOD, one of the primary antioxidant enzymes, catalyzes the conversion of superoxide anions into hydrogen peroxide and molecular oxygen [34]. GSH-Px is another essential antioxidant enzyme that catalyzes the reduction of hydrogen peroxide and lipid hydroperoxides into water and corresponding alcohols through the glutathione/glutathione disulfide redox cycle [35]. Therefore, the activities of SOD, GSH-Px, and other antioxidant enzymes are widely used to evaluate cellular antioxidant capacity and resistance to oxidative stress.

**Figure 8:**
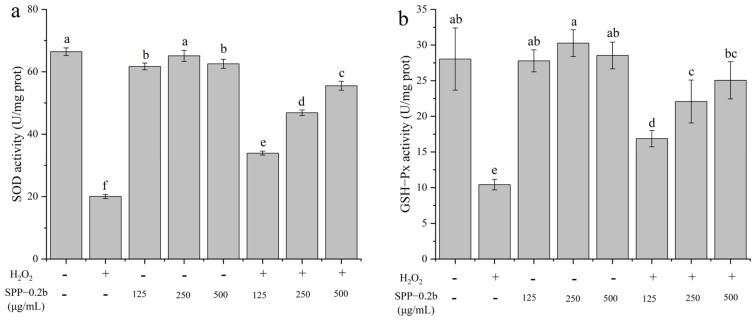
SPP-0.2b reverses H_2_O_2_-induced suppression of (**a**) SOD and (**b**) GSH-Px activities in Caco-2 cells. Data are shown as mean ± SD (*n* = 3). Different letters represent significant difference (*p* < 0.05).

Previous studies have demonstrated that H_2_O_2_ exposure suppresses the antioxidant defense system by reducing the activities of SOD and GSH-Px [24]. Consistent with these findings, our results showed that treatment with 250 μmol/L H_2_O_2_ decreased SOD and GSH-Px activities by 69.85% and 62.83%, respectively, relative to the untreated control (Figure 8). However, pretreatment with SPP-0.2b significantly restored antioxidant enzyme activity in a dose-dependent manner. Specifically, pretreatment with 125, 250, and 500 μg/mL SPP-0.2b increased SOD activity to 1.69-, 2.34-, and 2.77-fold of that in the H_2_O_2_-treated group, respectively (Figure 8a). Similarly, GSH-Px activity was elevated to 1.62-, 2.12-, and 2.40-fold of that in the H_2_O_2_-treated group following pretreatment with 125, 250, and 500 μg/mL SPP-0.2b, respectively (Figure 8b).

These findings indicate that enhancement of the antioxidant defense system may contribute to the intestinal barrier protective effect of SPP-0.2b against H_2_O_2_-induced disruption.

## 4. Conclusions

In summary, a polysaccharide fraction, designated SPP-0.2b, was isolated and purified from the pomace of *Hippophae rhamnoides* subsp. *sinensis*. SPP-0.2b possessed a weight-average molecular weight (Mw) of 42.405 kDa and was mainly composed of Rha, Ara, Gal, Man, and GalA. SPP-0.2b exhibited protective effects against H_2_O_2_-induced intestinal barrier dysfunction in Caco-2 monolayers, as evidenced by increased TEER value and reduced basolateral FITC-dextran fluorescence intensity following pretreatment. The protective effects of SPP-0.2b were associated with enhanced expression of TJ proteins and attenuation of oxidative stress. Specifically, SPP-0.2b reduced intracellular ROS and MDA accumulation while restoring the activities of the antioxidant enzymes SOD and GSH-Px. Collectively, these findings provide experimental evidence supporting further investigation of SPP-0.2b as a potential functional food ingredient for intestinal health. However, the present study has several limitations. Although the molecular weight and monosaccharide composition of SPP-0.2b were successfully characterized, these data alone are insufficient to establish a reliable structural model. More comprehensive structural analyses, including glycosidic linkage analysis and NMR spectroscopy, are required to further elucidate the fine structure of SPP-0.2b. In addition, the present findings were obtained using a single in vitro cell model under one oxidative stress condition. Therefore, further validation using additional experimental models, particularly in vivo studies, is required before the practical application of SPP-0.2b can be considered.

## Data Availability

The original contributions presented in the study are included in the article and Supplementary Materials; further inquiries can be directed to the corresponding author.

## Supplementary Materials

The following supporting information can be downloaded at: https://www.mdpi.com/article/10.3390/foods15152591/s1, Figure S1: Effect of SPP-0.2b on viability of Caco-2 cells.

## Abbreviations

The following abbreviations are used in this manuscript:

TJ	Tight junction
ROS	Reactive oxygen species
ZO-1	Zonula occludens-1
HPGPC	High-performance gel permeation chromatography
H_2_O_2_	Hydrogen peroxide
MEM	Minimum essential medium
FBS	Fetal bovine serum
MDA	Malondialdehyde
SOD	Superoxide dismutase
GSH-Px	Glutathione peroxidase
CCK-8	Cell Counting Kit-8
TEER	Transepithelial Electrical Resistance
FITC	Fluorescein isothiocyanate
PMSF	phenylmethylsulfonyl fluoride
PVDF	polyvinylidene difluoride
TBST	Tris-buffered saline with Tween 20
ECL	Electrochemiluminescence
Rha	Rhamnose
Ara	Arabinose
Gal	Galactose
Man	Mannose
GalA	Galacturonic acid
