# Supplementary material for: A Sea Buckthorn Pomace Polysaccharide Mitigates H2O2-Induced Impairment of Intestinal Barrier Integrity in Caco-2 Monolayers
Source: Foods. 2026 Jul 24;15(15):2591. doi: 10.3390/foods15152591 (PMC13465015; doi:10.3390/foods15152591)
Supplement: Supplementary file 1 [file foods-15-02591-s001.zip › foods-4393791-supplementary.pdf]

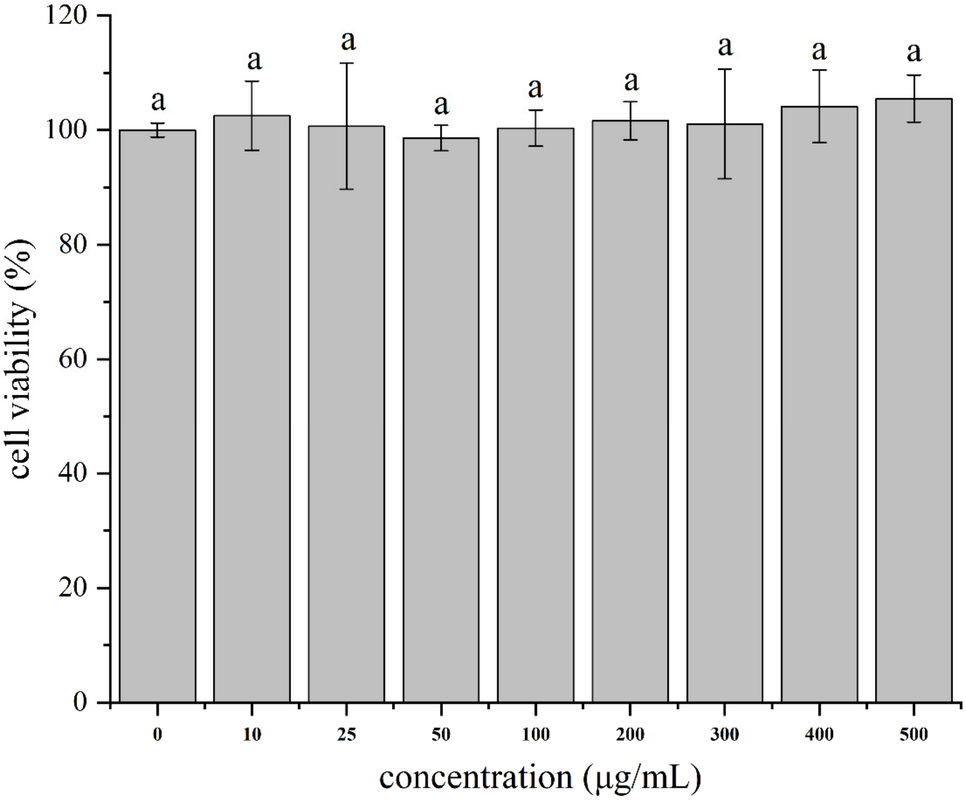


**Figure S1.** Effect of SPP-0.2b on viability of Caco-2 cells.
